# Stability of Dibromo-Dipyrromethene Complexes Coordinated with B, Zn, and Cd in Solutions of Various Acidities

**DOI:** 10.3390/molecules27248815

**Published:** 2022-12-12

**Authors:** Iuliia Aksenova, Vladimir Pomogaev

**Affiliations:** Laboratory of Photophysics and Photochemistry of Molecules, Department of Physics, National Research Tomsk State University, 634050 Tomsk, Russia

**Keywords:** dipyrromethene complexes, spectroscopy, protonation, stability of complexes, TD-DFT analysis

## Abstract

The spectral luminescent properties of dipyrromethenates halogenated with bromine on both ends of the long axis and coordinated using boron fluoride, zinc, or cadmium in neutral ethanol and acidified with hydrochloric acid solutions were studied. The constants of the acid–base equilibrium of the complexes in the proton-donor solvents in the ground and excited states was determined. The mechanisms of complex protonation were discussed, depending on the structure of the compounds. The electronic structures of the neutral and protonated compounds were modeled and analyzed based on the quantum-chemical method. The structures and spectral-luminescence properties were calculated using the SMD model of ethanol solvent using the TD-DFT theory with the B3LYP functional and the composite def2-SVP/def2-TZVP/def2-TZVPP_ECP basis sets, depending on the atomic number of the elements.

## 1. Introduction

The chemistry of dipyrromethene (dpm) compounds has been widely developed due to the existing variety of their various metal complexes, which have outstanding spectral and photophysical characteristics. Covalent dpm complexes with cations of p-, d-, and f-elements exhibit high chromophore activity in the visible region of the electromagnetic spectrum. Efficient fluorescence yields and high photostability of dpm complexes with boron fluoride BODIPY have been established [1,2,3,4]. The complexes of d-elements with two dpm ligands have been synthesized relatively recently. They also exhibit efficient and stable luminescence, with a tunable emission wavelength. The spontaneous coordination of these metal complexes enables them to construct self-assembled nanoarchitectures, such as supramolecules, low-dimensional nanostructures, and metal–organic frameworks [5,6,7,8].

The specific spectral-luminescence and photochemical properties allow for predicting and designing the broad avenue of materials based on dpm compounds in their practical use as active components of various optical devices: fluorescent probes [9,10,11], optical sensors [12,13,14], and photosensitizers for the generation of singlet oxygen [15,16,17]. Moreover, the high sensitivity of spectral-luminescent characteristics to changes in the structure of the ligand and the properties of the medium makes them very promising fluorescent pH sensors [18,19,20]. Moreover, potential applications of various dipyrromethene metal complexes, such as for catalysis and thermoelectric and photoelectric conversion, are widely discussed [7,21]. For successful creation of such novel optical materials, the information on their chemical stability under the operating conditions of a particular device in various environments, including at different acidity, is required.

Despite the large number of works describing the synthesis, there is a lack of published studies devoted to a detailed analysis of the influence of electronic, structural, and solvation factors on the stability and photo-induced properties of dpm and their metal complexes. The manifested properties have only recently begun to be analyzed [22,23,24]. The practical use of complex organic molecules—in particular, dpm compounds—requires deep study and an understanding of their inherit physical and chemical properties exhibited under different conditions; therefore, this is the most important step in designing such optical devices. The great practical importance is to establish the interaction between the complexing agent, ligand, and solvating medium influents on the associative, acid–base, and coordination properties of dpm. This knowledge could make the purposeful synthesis of metal complex structures with the desired characteristics and the subsequent creation of optical devices based on dpm compounds both easier and cheaper.

Thus, our research was aimed at studying the protonation processes of bromine substituted dipyrromethene complexes with various complexing agents such as metalloid B vs. transition metals Zn and Cd in regards to the structural stability and photophysical properties of these compounds in neutral and acidic solvents. The information about the features of protonation becomes important in the practical use of these complexes, namely, the incorporation of dyes into solid-state polymer matrices because the polymerization process often proceeds at low pH, i.e., in acidic media, which certainly affects the spectral and luminescent properties of the materials. In order to establish the influence of neutral and acidified environments, the dpm complexes, both in the ground and in the excited state, were investigated for a practical assessment of their stability in proton-donor media. The obtained values of the acid–base interaction constants and the revealed patterns of the influence of structural and solvation factors will allow us to optimize the search for leading compounds with the most successful combination of spectral and photophysical characteristics in dpm and their metal complexes.

## 2. Results

### 2.1. Experimental Stability Study

It has been established that the mechanism of proteolytic dissociation of BODIPY complexes includes several stages [25,26,27]. The initial step of proton binding to the fluorine atoms of BF_2_-dpm [BF_2_L] makes the B-N and B-F coordination bonds weaker. The weakening leads to their cleavage, with proton attaching to the pyrrole nitrogen that forms the protonated ligand H_2_L^+^ through one isosbestic point. As was noted in these publications, the isosbestic point indicates that the third chromophore, the molecular form of HL, does not accumulate in solution at recorded concentrations. However, it should be borne in mind that this is typical only for boron fluoride dpm complexes. For other dpm metal complexes, a change in the “classical” protonation mechanism, i.e., that the coordination center is not modified in the first place [22], should be assumed. Moreover, it has been hypothesized [28] those electronegative substituents, such as heavy bromine atoms in the structure of the studied molecules, can pull the electron density onto themselves, which should essentially reduce the proton attack on the coordination center and even change the known mechanism of the structural dissociation. Nevertheless, protonation occurs in any case, which is manifested in the registration of the electronic absorption and fluorescence spectra.

**Figure 1:**
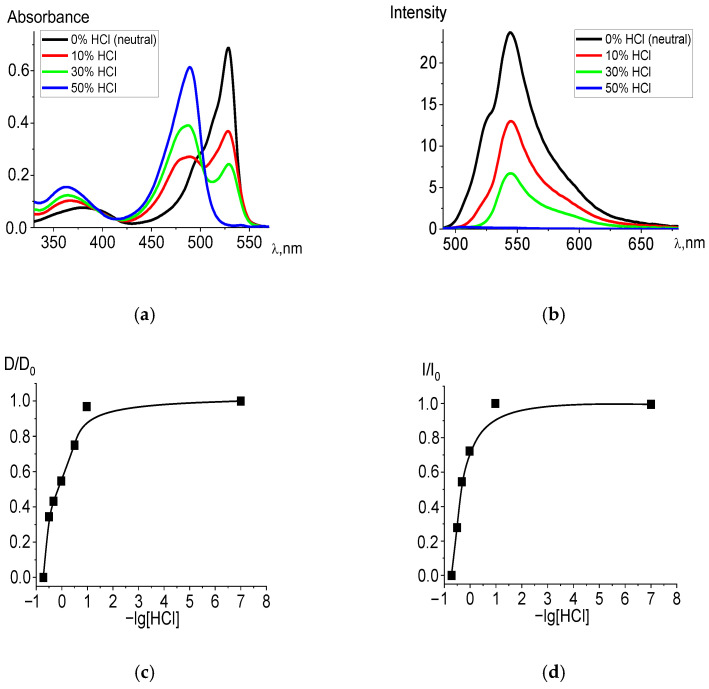
Changes in the absorption (**a**) and fluorescence (λ_ex_ = 470 nm) (**b**) spectra of Br_2_(CH_3_)_4_BODIPY in ethanol upon the addition of 36% (11.6 M) HCl, in varying amounts. Experimental titration curves for the ground state S_0_ (λ = 528 nm) (**c**) and excited state S_1_ (λ = 544 nm) (**d**).

The experiment showed that in acidified ethanol solutions, the studied dpm complexes entered into an exchange reaction with hydrochloric acid. The electronic absorption spectra of the products corresponded to the spectra of the protonated structures formed in the exchange reactions of the ligands, with the presence of an isosbestic point (500 nm) in the patterns of irreversible spectral transformations (Figure 1a). Table 1 shows the blue spectral shifts to shorter wave-length segment and values of −lg[HCl]_50_ in the corresponding state of the acidified ethanol solutions for BF_2_, Zn, and Cd diBr-substituted dpm complexes. The trend is supported by the TD-DFT calculations (see details in Section 2.2) of the compounds, as well as their HCl acidified forms in the ethanol. Moreover, the measured and calculated wavelengths are very similar for all the complexes in the acidic solvent, whereas the absorption time of BODIPY in a neutral environment is significantly longer than that in the cases of the remaining systems, based on the transition elements. Among the studied compounds, Br_2_(CH_3_)_4_BODIPY is the most stable in the ground state and dissociates only with a significant addition of acid (at the highest acid concentrations) in the solution. Nevertheless, protonation occurs in any case, which is manifested in the registration of the spectra of acidified ethanol solutions. Based on the spectral shifts, the −lg[HCl]_50_ values for the Franck–Condon state S_1_ were determined, from which it follows that the stability of the excited BODIPY complex is higher than that in the ground state, which is consistent with the high photostability of BODIPY-based laser media.

Changes are also observed in the fluorescence spectra (Figure 1b). Complete data for changes in the spectra are presented in Figure S1. When the ligand passes into the protonated form, the fluorescence intensity decreases due to a decrease in the concentration of the BODIPY fluorophore and an increase in the proportion of protonated H_2_L^+^ ligands formed, the fluorescence quantum yield of which does not exceed 0.003.

**Figure 2:**
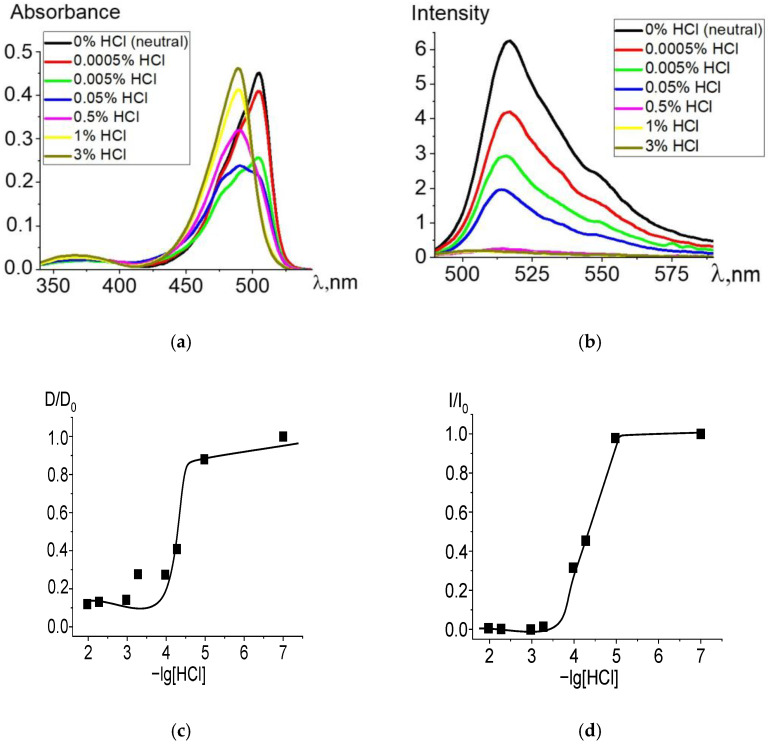
Changes in the absorption (**a**) and fluorescence (λ_ex_ = 460 nm) (**b**) spectra of Zn[Br_2_(CH_3_)_4_dpm]_2_ in ethanol upon the addition of 36% (11.6 M) HCl, in varying amounts. Experimental titration curves for the ground state S_0_ (λ = 504 nm) (**c**) and excited state S_1_ (λ = 515 nm) (**d**).

**Figure 3:**
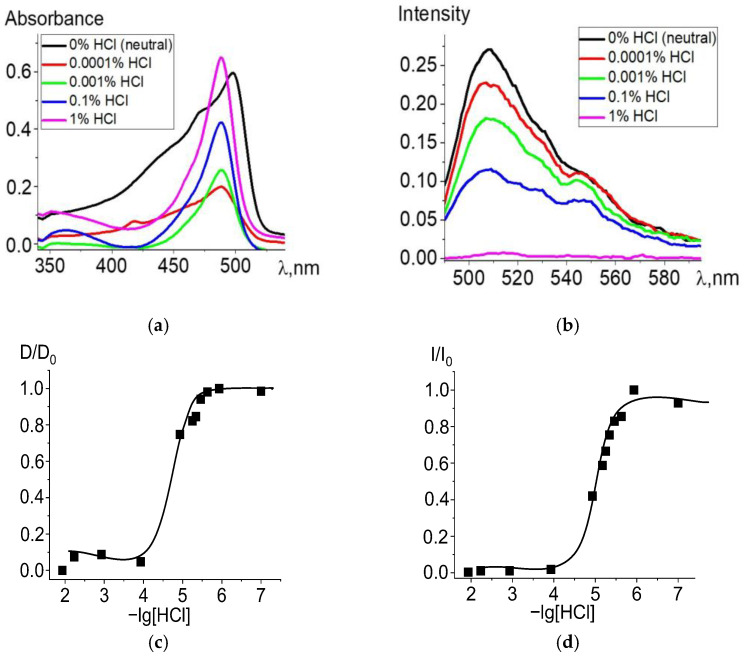
Changes in the absorption (**a**) and fluorescence (λ_ex_ = 470 nm) (**b**) spectra of Cd[Br_2_(CH_3_)_4_dpm]_2_ in ethanol upon the addition of 36% (11.6 M) HCl, in varying amounts. Experimental titration curves for the ground state S_0_ (λ = 498 nm) (**c**) and excited state S_1_ (λ = 508 nm) (**d**).

When ethanol solutions are acidified, Zn and Cd dpm complexes also enter into irreversible exchange reactions with hydrochloric acid. The degree of conversion of complexes into intermediate and final products of the proteolytic dissociation processes clearly reflects changes in the electronic absorption and fluorescence spectra (Figure 2 and Figure 3). Complete data for changes in the spectra are presented in Figures S2 and S3. The intense band of the Zn or Cd complex transforms into a spectrum formed by the protonated ligand Hdpm and the salt with the mineral acid HCl under increasing concentrations of hydrochloric acid in the solution, leading to the significant short-wavelength shift of the absorption maxima (Figure 2a and Figure 3a). The single intersection point at 495–500 nm of absorption spectra formed in solutions of neutral or low HCl concentration vs. those of high acidity is observed and assumed as an isosbestic point. Thus, only two stable chromophore forms are confirmed by the existence of some transition point between the spectra of dpm metal complexes in neutral ethanol and their protonated forms in acidified solution.

The attribution of absorption in the acidic solution to the protonated ligand is confirmed by the coincidence of the spectra of the acidified solutions of metal dpm (Zn and Cd) and BF_2_ in the corresponding complexes with the structurally identical ligands: 488–489 nm for Br_2_(CH_3_)_4_BODIPY, Zn[Br_2_(CH_3_)_4_dpm]_2_, and Cd[Br_2_(CH_3_)_4_dpm]_2_. At the same time, the absorption spectra of the corresponding neutral dpm differ significantly (by 22–28 nm) (Table 1).

In our case, it should be taken into account that the introduction of bromine atoms increases the stability of the complexes. It can be assumed that, due to the high electronegativity of bromine atoms, there is a decrease in the electron density in the coordinated nitrogen atoms and accordingly, in their basicity, which makes it difficult for the proton to attack the coordinated nitrogen atoms in the initial, limiting process of the proteolytic dissociation of these metal complexes. As a result, the described effect dominates over the opposite effect of the weakening of the Me-N coordination bonds, caused by the same structural factor, i.e., the outflow of electron density to the bromine atoms.

Based on the dependence obtained by Foerster, the −lg[HCl]_50_(S_1_)^F-C^ values for the S_1_ Franck–Condon states were determined (Table 1). It follows from the table that complexes with Zn and Cd in proton-donor media are less stable (they decompose at a lower acid concentration), both in the ground state and in the excited S_1_^F-C^ states, compared with the BODIPY complex.

Changes during protonation are also observed in the fluorescence spectra (Figure 2b and Figure 3b): fluorescence intensity of the protonated ligand decreases due to an increase in the proportion of protonated nearly nonfluorescent ligands formed with an insignificant maximum short-wavelength shift, in accordance with changes in absorption. Upon transition to the fluorescent S_1_ state, the stability of the protonated form slightly decreases due to the influence of the equilibrium solvation shell, which stabilizes the change in the electron density at the bonds with the complexing agent and reduces the efficiency of the proton addition to the dpm molecule.

### 2.2. Quantum Chemical Calculations and Theoretical Analysis

A high accuracy of the calculated spectral-luminescence properties of the isolated halogen–dpm compounds coordinated with BF_2_, Zn, and Cd was achieved in our previous theoretical description and the explanation of the observed photo-induced processes [24,29]. The same TD-DFT settings for optimization in both the ground and fluorescence states, as well as for calculating the long wavelength regions of absorption and emission spectra of the brominated compounds in neutral and HCl acidified forms in the ethanol solvent model were used in the present research (Table 2). This led to a perfect agreement with the measured fluorescence (Figure 1, Figure 2 and Figure 3). The electronic density (ED) of two identical dpm fragments of the Zn an Cd compounds provides the vanishing intensity of the longest wavelength band (544, 531 nm, respectively), which corresponds to the lowest energy dark state. The next higher bright state is nearly the same for both complexes (508, 507 nm), while Br_2_(CH_3_)_4_BODIPY exhibits an intensive first band at 528 nm, which determines high fluorescence efficiency vs. the Zn and Cd complexes.

**Figure 4:**
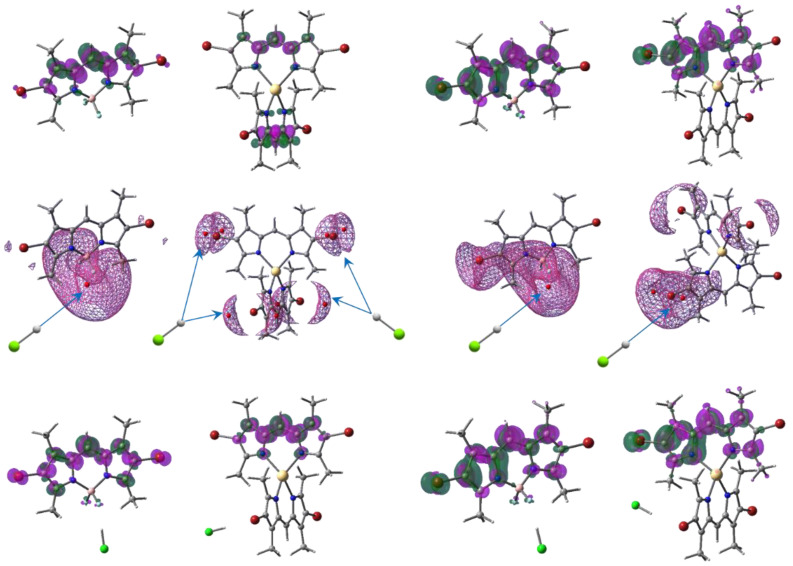
The uppermost pictures represent the ED redistribution (isodensity 2 × 10^3^ e^−^/bohr^3^) from the green to the magenta areas for the Br_2_(CH_3_)_4_BODIPY and Cd[Br_2_(CH_3_)_4_dpm]_2_ absorption (left side) and their emission (right side) in neutral ethanol. Global ESP minima (red circles) inside the mesh isolines 0.44 eV, with preferable directions of proton attack, are presented below. The bottom images are the same as the uppermost ones, but for the acidified (HCl) compounds.

The two lowest states show the same symmetry of ED localization on both fragments, forming a dissipative channel of internal conversion between the bright and dark states preferable over emission, leading to very low quantum harvest of fluorescence from the higher state (Figure 4 and [29]). On the other hand, despite the small oscillator strength of the dark state, it contributes an intensity to the emission spectra such that the spectral feature corresponds to the observed very weak emission intensities of the Zn and Cd systems (Figure 2 and Figure 3), with the peak at 515 and 508, respectively, as well as the small hill near 550 nm for both compounds, recognizable on the spectral manifolds. 

The mechanism of losing emission intensity is simpler for the BF_2_-based compounds, which exhibit very efficiency fluorescence, but halogenation, particularly with Br, quenches the process due to increasing nonradiative intersystem spin crossing from the lowest excited photoactive singlet to the triplet system [29]. The calculated longwave absorptions for all the compounds in the neutral solvent are blueshifted about Δλ_abs_ = 40 nm relative to the measured spectra (Table 1), which is an acceptable accuracy in comparison with the previous TD-DFT results, as previously discussed [24,29].

The experimental and theoretical values show the same trend of spectrum shortening from lighter to heavier complexing elements, but the energy gaps between systems based on B and Zn are essentially wider than those between Zn and Cd (Table 1 and Table 2). The calculations predict the lowest dark states of the Zn and Cd compounds, which are not observed on the measured spectra. The wavelengths of these transitions are comparable with Br_2_(CH_3_)_4_BODIPY in the range of 472–487 nm, while the next absorption intensive bands of the Zn and Cd compounds are 462 nm and 464 nm, respectively.

Br_2_(CH_3_)_4_BODIPY, based on the metalloid, is much more stable than the complexes of two dpm fragments coordinated with the transition metals M[Br_2_(CH_3_)_4_dpm]_2_ (M = Zn, Cd), and the dissociation mechanisms are different. The fact that the halogen substituents partially pull the electron density protonation of complexing BF_2_ remains the main reason for BODIPY dissociation, whereas the case of the Zn and Cd are more complicated because their low stability depends on weaker metal bonds of the transition elements with two dpm fragments, although the protonation of the halogenated ends does not make the bonds stronger.

The most preferred points of the metalloid complex in the ground and excited states for protonation are near the strongly negatively charged two fluorine atoms (Figure 4) at the global minima (−1.65 eV and −1.60 eV, respectively). All electrostatic potential (ESP) minima were calculated using MultiWFN [30]. The vicinities of the complexing transition elements are protected by dpm fragments, with methyl groups oriented almost perpendicular to each other. Such a configuration allows the ESP minima to be equivalently located only near the halogenated ends for all bromines (−0.59 eV) of both M[Br_2_(CH_3_)_4_dpm]_2_ in the ground state because of the nearly C_2v_ symmetry of the compounds (Figure 4, Figure S1 and Figure S2, Tables S2–S7). The symmetry is distorted significantly in the excited state [29], providing different ED’s on the dpm ends and the nearby ESP minima. The deepest minimum (−0.84 eV and −0.82 eV) was chosen to study the HCl acidification of the excited Zn and Cd complexes, respectively, and one of the minima in ground state. Only the interaction HCl with F_2_ was considered in both the ground and excited states of Br_2_(CH_3_)_4_BODIPY because this is the main method of dissociation.

The protonation of the brominated ends can only make a quantitative, rather than a qualitative, contribution, where the ESP minima are −0.44 eV for the ground states and −0.65 eV in the fluorescent conformation. It could be also noted that the difference between the measured and calculated results is less for the acidified compounds Δλ_abs_ = 30 than for the neutral solvent because of slight distortion symmetry (Table 1).

Acidification scarcely changes the fluorescence wavelengths (Table 2) of all the compounds, which coincides with the measure values, while absorption undergoes the blue spectral shift at less than Δλ_abs_ = 10 nm for M[Br_2_(CH_3_)_4_dpm]_2_, which is significantly stronger than that of Δλ_abs_ = 30 nm in Br_2_(CH_3_)_4_BODIPY. Moreover, the last system demonstrates a clear increase in the dissociated product with an increase in acidity, in comparison with the rest of compounds, where complexing center is protected from proton attack.

## 3. Materials and Methods

**Figure 5:**
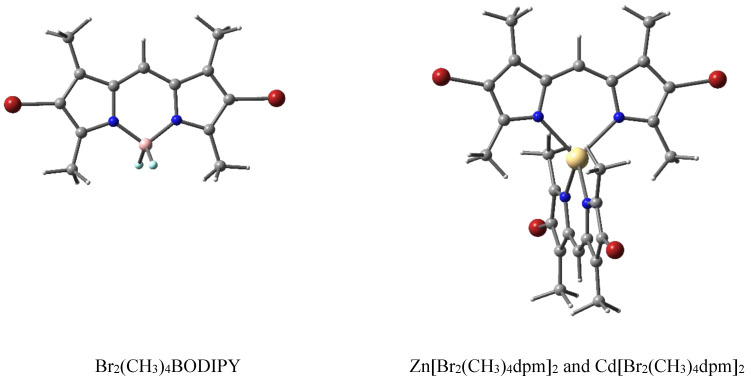
Structural formulas of dipyrromethene complexes, where the atoms are represented with the following colors: C (gray), N (blue), and H (white), halogenated with Br (red) and coordinated using both Zn and Cd (yellow) or B (pink), terminated by F (light blue).

The presented dpm complexes were synthesized at the G.A. Krestov Institute of Solution Chemistry of the Russian Academy of Sciences (Ivanovo). The descriptions of the synthesis and control of the purity of the structure using thin-layer chromatography and IR spectroscopy are described in [31,32,33]. Structural formulas of 3,3′,5,5′-tetramethyl-4,4′-dibromo-2,2′-dipyrromethene complexes coordinated with BF_2_, Zn, and Cd, which are abbreviated in this work as Br_2_(CH_3_)_4_BODIPY, Zn[Br_2_(CH_3_)_4_dpm]_2_, and Cd[Br_2_(CH_3_)_4_dpm]_2_, respectively, are shown in Figure 5. Full chemical structures and designations are provided in the Supplementary Materials (Table S1).

The studied complexes differ in the complexing agents (BF_2_, Zn, Cd) and contain heavy Br atoms located on the periphery of the dpm core in the β-position, i.e., all dpm fragments halogenated with Br on both ends of the long axis. Undried ethanol was used as a solvent. The solutions were acidified using a high-purity 36% aqueous HCl (11.6 M).

The absorption spectra of the studied compounds were recorded on a Cary5000 spectrometer (Varian) in a quartz cell with an optical path length of 1 cm under standard conditions. The spectra of fluorescence were measured using a Cary Eclipse spectrometer (Varian) at room temperature (298 K). The typical concentration of the investigated compounds was 10^−5^–10^−6^ M.

To determine the quantitative characteristics of the dye’s stability by spectrophotometric titration, a series of water–ethanol solutions of dpm complexes with a successively changing content of hydrochloric acid were studied. After adding the acid, the solutions were held for up to 180 min to establish equilibrium, which was confirmed by the invariance of the absorption spectra.

Taking into account the results of the work in [34,35], which discussed the mechanisms of protonation of such compounds, to assess the stability of dpm complexes, we chose the value −lg[HCl]_50_. In this case, the concentration of the complexes decreases due to their decomposition by 50% with the formation of a protonated ligand and the release of the complexing agent from the complex. The titration curves were obtained as dependences of the relative change in absorption at the chosen wavelength ΔD/D_0_ or fluorescence ΔI/I_0_ on −lg[HCl]_50_. The lower the −lg[HCl]_50_ value, the higher the stability of the complexes in the proton-donor media, i.e., the higher the acid concentration required to achieve 50% conversion of the complex to a protonated ligand according to the classical definition [36,37,38].

In the case of the acid titration of neutral molecules with a pronounced proton acceptor center, the value of −lg[HCl]_50_ can be used to estimate the pKa value, which characterizes the basicity of the molecule and is proportional to the efficiency of proton abstraction from the acid conjugated to a given base [39]. In this regard, by analogy with the basicity characteristics, the value of −lg[HCl]_50_ (S_1_^F-C^) in the excited Franck–Condon states of S_1_ was estimated from the shifts of the absorption bands from the neutral complex to the protonated ligand due to the Foerster relation [40,41].

In order to reveal the spectral-luminescent features of the dpm complexes and their acidified forms, electronic structures were optimized in both the ground and fluorescence states, and the photophysical properties were calculated in the continuum of the solvation model using the electron density (SMD) [42] of the ethanol solvent by means of the hybrid B3LYP [43,44] exchange-correlation function implemented in Gaussian 16 [45] of the TD-DFT method, in combination with the composite def2-SVP[H, B, C, N, F]/def2-TZVP[Br, Zn]/def2-TZVPP_ECP[Cd] basis sets for different elements of the compounds, as successfully used in a recent study [24,29].

## 4. Conclusions

The experimental and theoretical competitive evaluation of the stability and photo-physical properties of 3,3′,5,5′-tetramethyl-4,4′-dibromo-2,2′-dipyrromethene complexes coordinated with Zn, Cd, and BF_2_ (Zn[Br_2_(CH_3_)_4_dpm]_2_, Cd[Br_2_(CH_3_)_4_dpm]_2_, Br_2_(CH_3_)_4_BODIPY) was carried out in the proton-donor media. The mechanisms of the dissociation of BF_2_ vs. Zn and Cd complexes in neutral and acidified ethanol solvents were confirmed by analyzing the titration curves and comparing the spectral data for both absorption and fluorescence in different hydrochloric acid concentrations. The theoretical assumptions about the protonation pathways were confirmed using quantum-mechanical calculations.

The electronic structures of the neutral and HCl acidified Br_2_(CH_3_)_4_BODIPY, Zn[Br_2_(CH_3_)_4_dpm]_2_ and Cd[Br_2_(CH_3_)_4_dpm]_2_ were optimized in the ground and fluorescent states to calculate their spectral-luminescence properties in the ethanol solvent SMD model using TD-DFT theory with the B3LYP functional and the hybrid def2-SVP[H, B, C, N, F]/def2-TZVP[Br, Zn]/def2-TZVPP_ECP[Cd] basis sets. The calculations exhibited the expected correlation between the measured and theoretical absorption spectra for the neutral, and better correlation for acidified, structures, as well as perfect agreement for fluorescence. The pathways of the protonation attacks were detected, based on ESP analysis. In addition, these results point to differences in the initial stages of proteolytic dissociation, depending on the nature of the complexing agent. In BODIPY complexes, the primary interaction occurs with the complexing agent BF_2_, but in the transition metal Zn and Cd complexes of dipyrromethenes, the most preferable direction for initial protonation is along the long axis of the dpm fragments between the brominated end and HCl from the solvent. Thus, it has been shown that the introduction of halogen atoms has a significant effect on the protonation process of the dipyrromethene metal complexes, engaging in interaction with the protons of the medium and screening the coordination centers of the molecules.

The obtained results show that the highest stability in the ground and excited states is typical for BODIPY. This complex is stable, even in strongly acidified media, while the metal complexes of dpm (Zn and Cd) are more likely to undergo dissociation in the proton donor solvents. In the future, it is necessary to develop approaches to increase the stability of the dpm metal complexes for their successful practical application in various optical devices.

## Figures and Tables

**Table 1:** Stability characteristics of the dpm complexes in proton-donor media for the ground (S_0_), Frank–Condon (S_1_)^F-C^, and fluorescence (S_fl_) states. Experimental/calculated * absorption (λ_abs_) wave-lengths (in parentheses), with oscillator strengths of the compounds in ethanol solvent, neutral and acidified, using hydrogen chloride (acidic).

Compound	λ_abs_, nm (Neutral)	λ_abs_, nm (Acidic)	−lg[HCl]_50_ (S_0_)	−lg[HCl]_50_ (S_1_)^F-C^	−lg[HCl]_50_ (S_fl_)
Br_2_(CH_3_)_4_BODIPY	528/528 (0.85)	489/488 (0.95)	−0.2	−3.2	−0.4
Zn[Br_2_(CH_3_)_4_dpm]_2_	504/504 (1.12)	489/485 (1.15)	4.3	3.1	4.3
Cd[Br_2_(CH_3_)_4_dpm]_2_	498/502 (1.12)	488/487 (1.15)	4.7	3.9	5.0

* Calculated numbers +40 nm for neutral and +30 nm for acidic conditions.

**Table 2:** Absorption (λ_abs_) and fluorescence (λ_fl_) wave-lengths with oscillator strengths (in parentheses) of the compounds in ethanol solvent neutral and acidified states using hydrogen chloride (acidic).

Compound	λ_fl_, nm; Neutral	λ_fl_, nm; Acidic	λ_abs_, nm; Neutral	λ_abs_, nm; Acidic
Br_2_(CH_3_)_4_BODIPY	528.3 (0.467)	528.9 (0.482)	487.5 (0.854)	451 (0.405)
Zn[Br_2_(CH_3_)_4_dpm]_2_	543.7 (0.001) 508.2 (0.343)	530.8 (0.002) 507.6 (0.356)	482.8 (0.001) 464.4 (1.122)	477.0 (0.001) 455.1 (1.145)
Cd[Br_2_(CH_3_)_4_dpm]_2_	532.3 (0.000) 507.4 (0.287)	521.5 (0.001) 504.6 (0.332)	472.3 (0.001) 461.8 (1.122)	473.0 (0.001) 456.5 (1.159)

## Data Availability

Not applicable.

## Supplementary Materials

The following supporting information can be downloaded at: https://www.mdpi.com/article/10.3390/molecules27248815/s1, Table S1: Structures and designations of dipyrromethene complexes; Figure S1: Changes in the absorption and fluorescence spectra upon protonation for the boron dipyrromethene complex; Figure S2: Changes in the absorption and fluorescence spectra upon protonation for the zinc dipyrromethene complex; Figure S3: Changes in the absorption and fluorescence spectra upon protonation for the cadmium dipyrromethene complex; Figures S4 and S5: The complexes calculated for absorption in the ground states; Tables S2–S7: The complexes calculated for fluorescence in the excited states.
