# Supplementary material for: Stability of Dibromo-Dipyrromethene Complexes Coordinated with B, Zn, and Cd in Solutions of Various Acidities
Source: Molecules. 2022 Dec 12;27(24):8815. doi: 10.3390/molecules27248815 (PMC9784619; doi:10.3390/molecules27248815)
Supplement: Supplementary file 1 [file molecules-27-08815-s001.zip › molecules-2099270-supplementary.pdf]

Supplementary information for

Table S1. Structures and designations of dipyrromethene complexes.

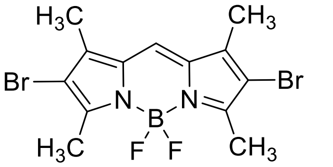	complexes of BF_2 with 3,3′,5,5′-tetramethyl-4,4′-dibromo-2,2′-dipyrromethene	$Br_2(CH_3)_4$BODIPY
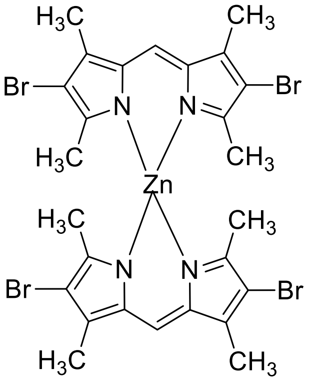	complexes of zinc(II) with 3,3′,5,5′-tetramethyl-4,4′-dibromo-2,2′-dipyrromethene	$Zn[Br_2(CH_3)_4dpm]_2$
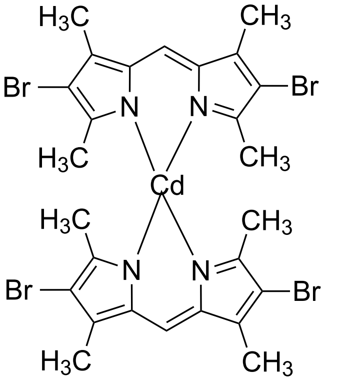	complexes of cadmium (II) with 3,3′,5,5′-tetramethyl-4,4′-dibromo-2,2′-dipyrromethene	$Cd[Br_2(CH_3)_4dpm]_2$

Figure S1. Changes in the absorption and fluorescence spectra upon protonation for boron dipyrromethene complex.

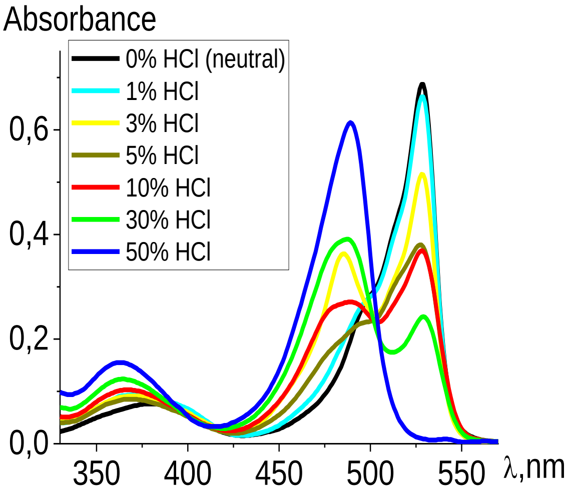	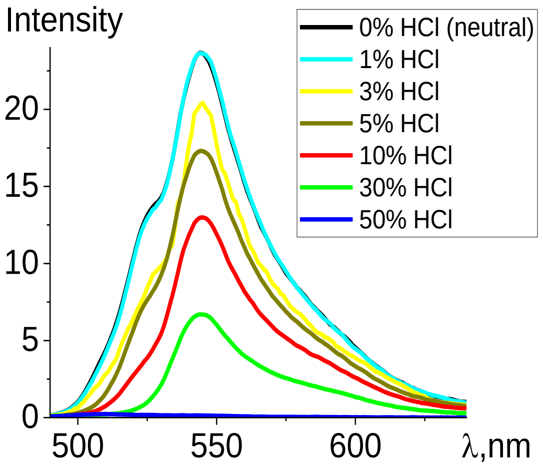
Changes in the absorption spectra of $Br_2(CH_3)_4$BODIPY in ethanol upon the addition of 36% (11.6 M) HCl with varying amounts.	Changes in the fluorescence (λ_{ex} = 470 nm) spectra of $Br_2(CH_3)_4$BODIPY in ethanol upon the addition of 36% (11.6 M) HCl with varying amounts.

Figure S2. Changes in the absorption and fluorescence spectra upon protonation for zinc dipyrromethene complex.

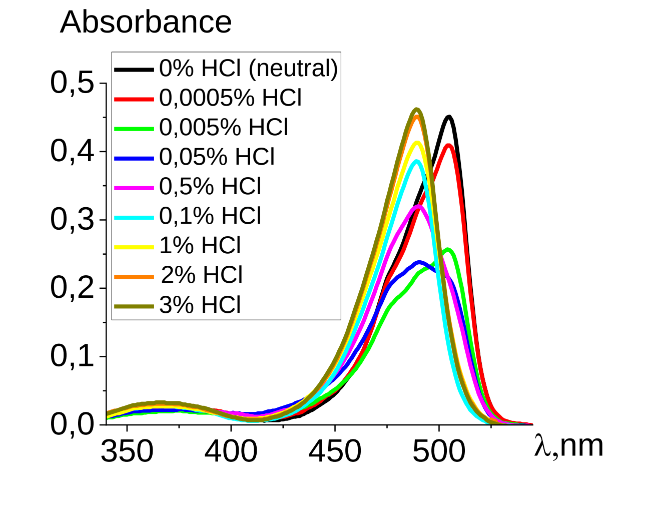

Changes in the absorption spectra of $Zn[Br_2(CH_3)_4dpm]_2$ in ethanol upon the addition of 36% (11.6 M) HCl with varying amounts.

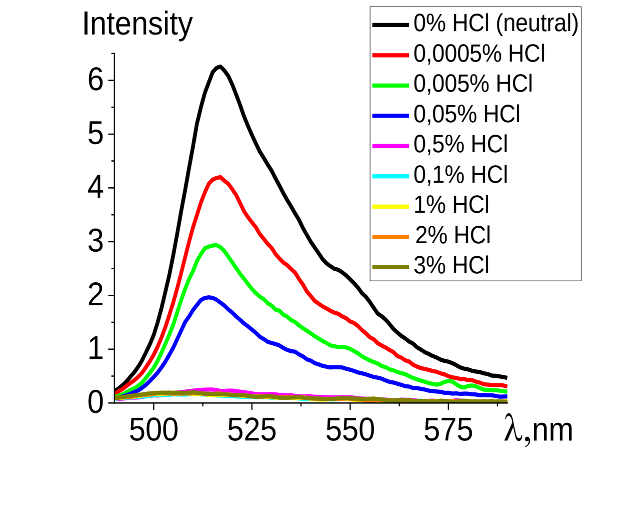

Changes in the fluorescence (λ_{ex} = 460 nm) spectra of $Zn[Br_2(CH_3)_4dpm]_2$ in ethanol upon the addition of 36% (11.6 M) HCl with varying amounts.

Figure S3. Changes in the absorption and fluorescence spectra upon protonation for cadmium dipyrromethene complex.

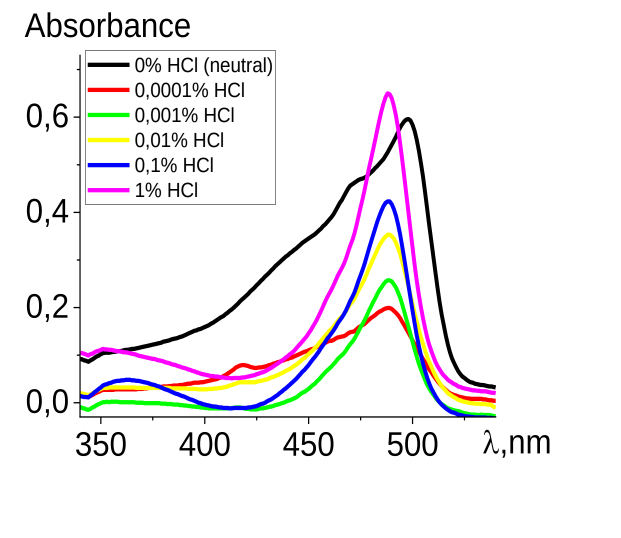

Changes in the absorption spectra of Cd[$Br_2(CH_3)_4$dpm]$_2$ in ethanol upon the addition of 36% (11.6 M) HCl with varying amounts.

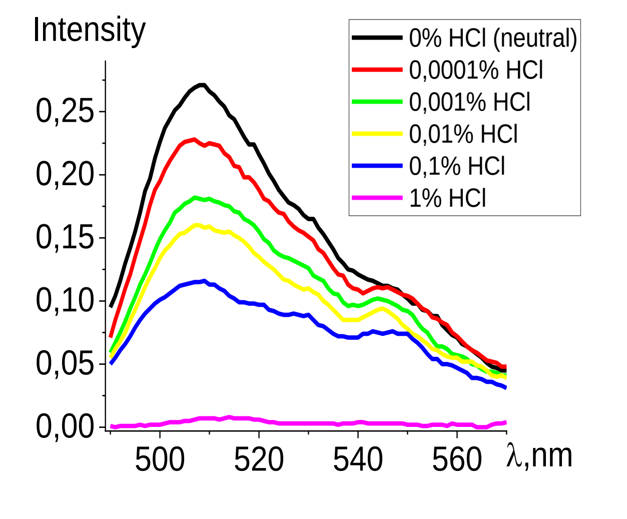

Changes in the fluorescence (λ_{ex} = 470 nm) spectra of Cd[$Br_2(CH_3)_4$dpm]$_2$ in ethanol upon the addition of 36% (11.6 M) HCl with varying amounts.

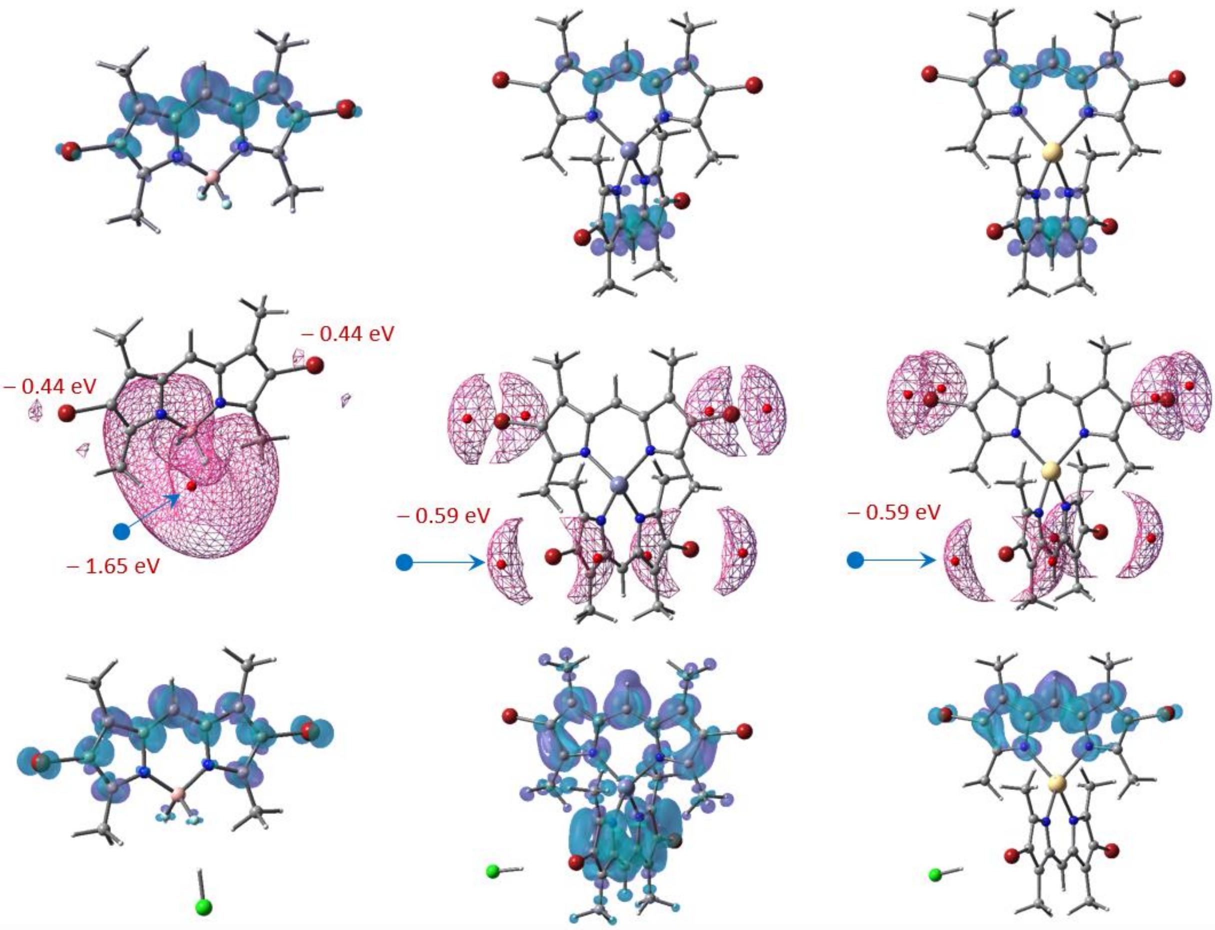

Figure S4. The complexes in ground states for absorption EDD(S_{ground}→S_{bright}). $Br_2(CH_3)_4$BODIPY, Zn[$Br_2(CH_3)_4$dpm$]_2$, and Cd[$Br_2(CH_3)_4$dpm$]_2$ are presented from left to right. The top line is EDD (isodensity 2×10^3 e^-/$bohr^3$) in neutral ethylene and bottom line is EDD in acidified (HCl) solvent with ED redistributes to purple area. Global ESP minima (large red and small white circles) inside mesh isolines 0.44 eV with preferable directions of proton (HCl) attack are illustrated with pictures on the middle line.

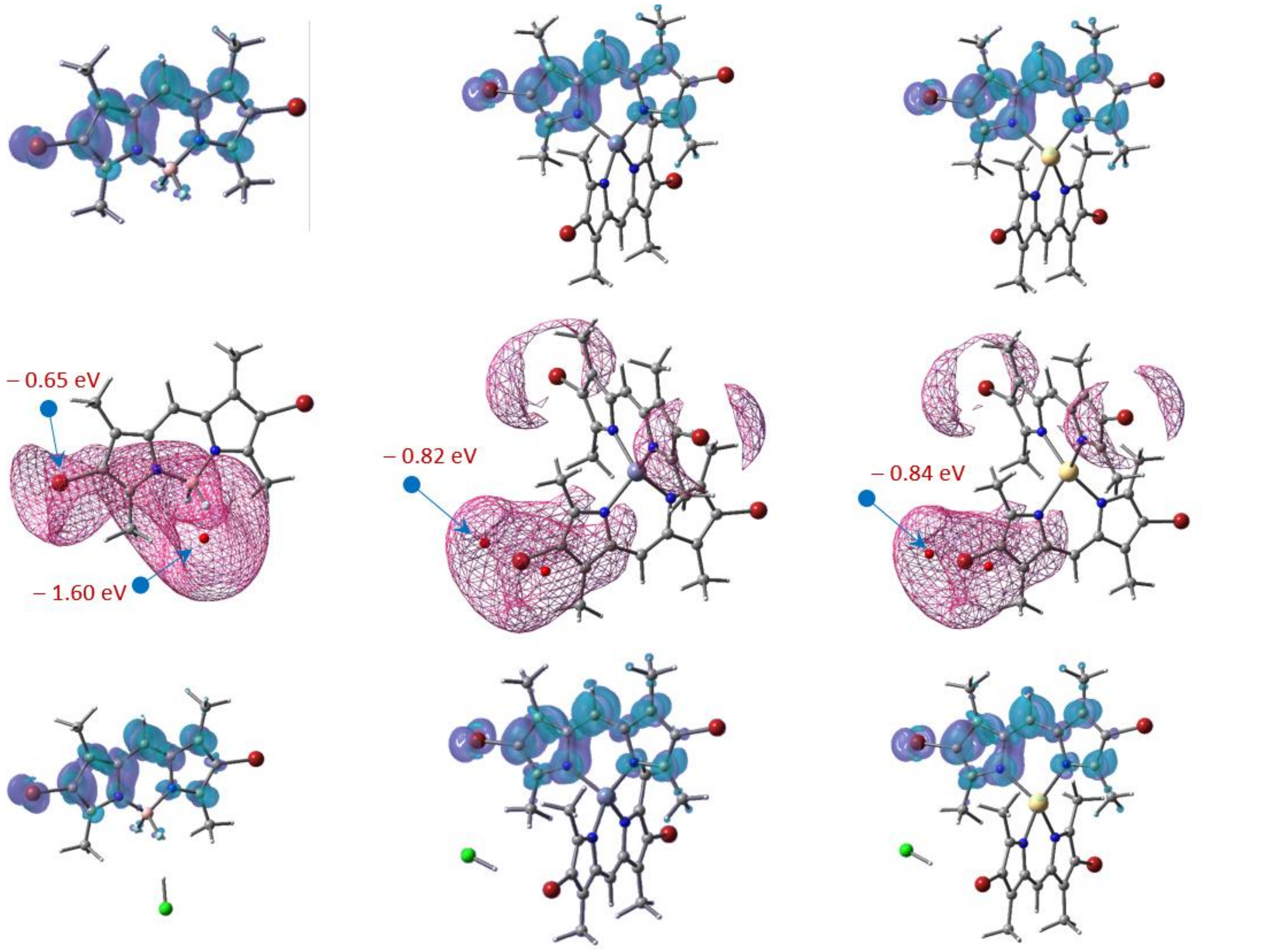

Figure S5. The complexes in excited states for fluorescence **EDD(S_{fl}→S_0)**. $Br_2(CH_3)_4$BODIPY, Zn[$Br_2(CH_3)_4$dpm$]_2$, and Cd[$Br_2(CH_3)_4$dpm$]_2$ are presented from left to right. The top line is EDD (isodensity 2×10^3 e^-/bohr^3) in neutral ethylene and bottom line is EDD in acidified (HCl) solvent with ED redistributes to purple area. Global ESP minima (large red and small white circles) inside mesh isolines 0.44 eV with preferable directions of proton (HCl) attack are illustrated with pictures on the middle line.

Table S2. Coordinates of $Br_2(CH_3)_4$BODIPY optimized structures in ground states for absorption

atom	neutral			acidic		
	X	Y	Z	X	Y	Z
B	0.000031	-1.327232	-0.018150	0.012720	-1.400485	0.184561
N	-1.257579	-0.405337	-0.007761	1.268492	-0.491002	0.107579
C	-2.548523	-0.802538	-0.012506	2.560895	-0.888968	0.121027
C	-3.361422	0.360177	0.000709	3.372807	0.269196	0.026844
C	-2.549435	1.496879	0.013863	2.561413	1.404660	-0.045104
C	-1.216993	0.989974	0.008245	1.229023	0.902048	0.005447
C	-0.000028	1.668392	0.016203	0.012320	1.579424	-0.031990
C	1.216964	0.990018	0.008602	-1.204194	0.903559	0.030983
C	2.549386	1.496972	0.014610	-2.536693	1.407853	0.008877
C	3.361418	0.360299	0.001695	-3.347842	0.273414	0.098197
C	2.548564	-0.802445	-0.011758	-2.535590	-0.885781	0.174902
N	1.257605	-0.405291	-0.007393	-1.243244	-0.489448	0.133905
F	-0.000119	-2.142824	1.109835	0.000640	-2.280483	-0.935558
F	0.000213	-2.116742	-1.164542	0.024328	-2.166605	1.348386
C	-2.966267	-2.230943	-0.028948	2.982856	-2.314079	0.191079
H	-2.571660	-2.742997	-0.920711	2.392661	-2.863863	0.938025
H	-2.571917	-2.763315	0.850950	2.830888	-2.811654	-0.782267
H	-4.060006	-2.319875	-0.030127	4.047046	-2.394317	0.448845
B	-5.250520	0.364549	0.000482	5.260770	0.267193	-0.001655
C	-2.973682	2.926448	0.030195	2.988479	2.829360	-0.150340
H	-3.590306	3.145875	0.918308	3.576956	2.998301	-1.068275
H	-2.112681	3.608707	0.038141	2.130920	3.515730	-0.165952
H	-3.590054	3.166184	-0.852828	3.634351	3.111162	0.698412
C	2.973576	2.926555	0.031065	-2.964098	2.833076	-0.087440
H	2.112549	3.608783	0.038757	-2.106237	3.518546	-0.118317
H	3.589931	3.146005	0.919359	-3.569102	3.003344	-0.994344
H	3.590200	3.166313	-0.851776	-3.594055	3.114909	0.773162
B	5.250516	0.364738	0.002024	-5.235992	0.274124	0.110141
C	2.966366	-2.230836	-0.028075	-2.957568	-2.310383	0.254473
H	4.060107	-2.319728	-0.028944	-4.018232	-2.389231	0.526633
H	2.571786	-2.763218	0.851715	-2.818475	-2.810890	-0.719241
H	2.572029	-2.742908	-0.919947	-2.357746	-2.858425	0.995104
H	-0.000049	2.759364	0.028713	0.012149	2.667119	-0.115427
H				-0.000792	-4.049923	-0.968003
Cl				-0.001742	-5.354033	-1.070261

Table S3. Coordinates of $Zn[Br_2(CH_3)_4dpm]_2$ optimized structures in ground states for absorption

atom	neutral			acidic		
	X	Y	Z	X	Y	Z
Zn	-0.000819	-0.000012	0.001406	-0.010875	0.013405	0.017783
N	-1.082865	-1.370623	-1.077048	-1.131892	-1.337125	-1.052169
C	-2.022491	-1.194536	-2.010835	-2.077012	-1.124277	-1.974154
C	-2.476636	-2.473472	-2.460828	-2.553165	-2.384177	-2.440905
C	-1.786653	-3.465282	-1.774783	-1.877760	-3.405503	-1.781703
C	-0.903075	-2.744655	-0.897282	-0.973232	-2.717280	-0.901257
C	-0.000980	-3.327600	-0.000175	-0.064602	-3.328682	-0.026607
C	0.900907	-2.745594	0.897752	0.862159	-2.771338	0.858751
C	1.784717	-3.467131	1.774269	1.749021	-3.507446	1.723169
C	2.474182	-2.476034	2.461870	2.459738	-2.527562	2.403925
C	2.019604	-1.196634	2.013650	2.019818	-1.241979	1.964254
N	1.080192	-1.371753	1.079463	1.066223	-1.400458	1.041388
N	1.081902	1.369571	-1.077760	1.027506	1.401059	-1.070475
C	2.021301	1.192579	-2.011598	1.950330	1.230481	-2.023384
C	2.476539	2.471073	-2.461755	2.376300	2.509604	-2.490371
C	1.787494	3.463552	-1.775746	1.687695	3.498502	-1.797690
C	0.903308	2.743782	-0.898146	0.832747	2.773906	-0.894405
C	0.001725	3.327616	-0.001111	-0.056592	3.344007	0.024139
C	-0.900681	2.746487	0.896870	-0.923467	2.747921	0.947762
C	-1.783914	3.468864	1.773268	-1.804276	3.447356	1.846039
C	-2.474252	2.478424	2.460950	-2.453410	2.439104	2.548689
C	-2.020813	1.198591	2.012821	-1.980164	1.173114	2.091457
N	-1.081178	1.372823	1.078684	-1.066109	1.370056	1.135292
Br	-3.809954	-2.721584	-3.784549	-3.901557	-2.607512	-3.757885
Br	3.807602	-2.725523	3.785227	3.797878	-2.819333	3.711294
H	-0.000766	-4.420819	-0.000961	-0.083952	-4.420683	-0.039769
Br	3.810058	2.717881	-3.785517	3.675932	2.786874	-3.839638
Br	-3.807408	2.729187	3.784332	-3.761375	2.679400	3.897035
H	0.002470	4.420835	-0.002008	-0.077005	4.436134	0.019663
C	-2.488424	0.143462	-2.475171	-2.522679	0.230374	-2.409425
H	-3.570171	0.271822	-2.300182	-1.983585	1.014211	-1.861072
H	-1.955651	0.947830	-1.950253	-2.347237	0.380210	-3.488407
H	-2.325047	0.268554	-3.559080	-3.603797	0.369111	-2.239389
C	-1.914636	-4.949951	-1.896699	-2.053885	-4.878239	-1.961499
H	-2.645041	-5.222507	-2.671910	-1.779059	-5.191898	-2.983567
H	-0.953298	-5.420259	-2.162130	-1.438279	-5.458018	-1.260245
H	-2.246679	-5.407528	-0.949556	-3.105178	-5.175512	-1.809148
C	1.913344	-4.951923	1.893995	1.875183	-4.988822	1.870043
H	0.951835	-5.423230	2.156982	1.536359	-5.321513	2.866928
H	2.247307	-5.407751	0.946674	1.285068	-5.533253	1.119931
H	2.642587	-5.225321	2.670004	2.925411	-5.309802	1.769018
C	2.485323	0.140885	2.479586	2.510830	0.086673	2.429046
H	2.327733	0.262302	3.564729	2.338316	0.217843	3.510947
H	3.565856	0.271999	2.298978	3.596961	0.188944	2.265718
H	1.948095	0.945774	1.960027	2.002147	0.901660	1.896703
C	2.486217	-0.145864	-2.475661	2.422370	-0.104867	-2.489268
H	3.567342	-0.276049	-2.298084	3.513749	-0.206916	-2.365312
H	1.950917	-0.949706	-1.952520	1.933321	-0.911797	-1.926999
H	2.325321	-0.270015	-3.560032	2.209424	-0.250751	-3.562077
C	1.916840	4.948095	-1.897762	1.820751	4.978222	-1.956366
H	2.646664	5.219935	-2.673771	1.693202	5.278049	-3.010087
H	0.955691	5.419397	-2.162087	1.080170	5.528928	-1.360005
H	2.250424	5.405295	-0.950972	2.822476	5.324865	-1.647379
C	-1.911220	4.953779	1.892883	-1.991196	4.922210	1.994891
H	-0.949489	5.424143	2.156766	-1.812616	5.243407	3.035168
H	-2.243859	5.409947	0.945264	-1.312987	5.494763	1.347350
H	-2.640899	5.227879	2.668234	-3.023862	5.220949	1.744262
C	-2.487559	-0.138487	2.479003	-2.406474	-0.175116	2.564162
H	-2.327391	-0.260906	3.563673	-2.269181	-0.277545	3.653692
H	-3.568744	-0.267715	2.301070	-3.477502	-0.347538	2.361229
H	-1.952934	-0.943906	1.957572	-1.829331	-0.965792	2.066261
H				-2.265328	-2.977674	-5.621962
Cl				-1.374710	-3.169095	-6.551505

Table S4. Coordinates of Cd[$Br_2(CH_3)_4$dpm]$_2$ optimized structures in ground states for absorption

atom	neutral			acidic		
	X	Y	Z	X	Y	Z
Cd	-0.000700	0.000000	-0.000068	0.030783	0.024941	-0.031750
N	-1.074519	-1.649437	-1.130811	-1.173001	-1.584130	-1.093458
C	-1.987041	-1.502719	-2.094425	-2.121046	-1.397534	-2.016467
C	-2.414397	-2.794369	-2.537245	-2.587689	-2.669657	-2.461001
C	-1.732669	-3.764500	-1.814775	-1.902930	-3.671945	-1.783094
C	-0.882868	-3.016030	-0.923374	-1.001855	-2.957788	-0.916554
C	-0.001585	-3.587285	0.000738	-0.095479	-3.558910	-0.031196
C	0.879868	-3.016050	0.924702	0.841869	-3.019677	0.852357
C	1.729445	-3.764600	1.816250	1.690765	-3.798218	1.721989
C	2.411438	-2.794536	2.538552	2.437999	-2.853951	2.410358
C	1.984530	-1.502840	2.095419	2.054444	-1.547263	1.966405
N	1.072000	-1.649464	1.131801	1.103097	-1.660950	1.038836
N	1.073381	1.648374	-1.132146	1.088460	1.708812	-1.098116
C	1.985693	1.500808	-2.095827	2.021258	1.591251	-2.048191
C	2.414352	2.792061	-2.538553	2.398098	2.892848	-2.495568
C	1.733679	3.762826	-1.815944	1.664889	3.841615	-1.792917
C	0.883142	3.015152	-0.924579	0.837058	3.067525	-0.902633
C	0.002487	3.587280	-0.000402	-0.068091	3.601184	0.023470
C	-0.879593	3.016922	0.923499	-0.912362	2.998115	0.960975
C	-1.728419	3.766270	1.815093	-1.787861	3.718580	1.852303
C	-2.411485	2.796845	2.537241	-2.417589	2.727890	2.592311
C	-1.985901	1.504752	2.094001	-1.937527	1.450651	2.158417
N	-1.073157	1.650523	1.130455	-1.039856	1.626382	1.187174
Br	-3.707166	-3.081714	-3.894614	-3.932605	-2.930806	-3.776666
Br	3.704051	-3.082014	3.896042	3.756575	-3.185339	3.731351
H	-0.001739	-4.680643	0.000872	-0.128035	-4.651289	-0.030912
Br	3.707385	3.078206	-3.895923	3.694651	3.242576	-3.832800
Br	-3.703830	3.085526	3.894732	-3.707801	2.972616	3.960206
H	0.003841	4.680638	-0.000167	-0.122487	4.692653	0.016972
C	-2.454495	-0.176464	-2.591023	-2.572967	-0.051556	-2.473311
H	-3.528708	-0.026290	-2.386429	-2.102535	0.738115	-1.869939
H	-1.894234	0.636741	-2.107729	-2.312097	0.122074	-3.531898
H	-2.323309	-0.087115	-3.682467	-3.667400	0.055599	-2.392015
C	-1.836156	-5.252834	-1.918048	-2.065503	-5.149666	-1.937522
H	-2.564730	-5.546482	-2.687426	-1.730417	-5.487790	-2.933889
H	-0.868438	-5.710867	-2.182790	-1.490548	-5.711105	-1.188244
H	-2.156685	-5.705727	-0.964872	-3.122945	-5.447090	-1.839428
C	1.832496	-5.252947	1.919771	1.723417	-5.287567	1.847335
H	0.864596	-5.710679	2.184361	0.802526	-5.674413	2.317018
H	2.153110	-5.706063	0.966727	1.814095	-5.777660	0.864568
H	2.560826	-5.546690	2.689345	2.571924	-5.614701	2.465570
C	2.452443	-0.176635	2.591719	2.596472	-0.239194	2.433515
H	2.320951	-0.086851	3.683092	2.406922	-0.088431	3.510182
H	3.526789	-0.027039	2.387417	3.689007	-0.183494	2.291943
H	1.892719	0.636670	2.107971	2.131969	0.590296	1.881422
C	2.451833	0.174124	-2.592522	2.545095	0.279664	-2.526905
H	3.525818	0.022745	-2.387618	3.639717	0.215746	-2.405728
H	1.890544	-0.638578	-2.109564	2.085942	-0.546176	-1.964491
H	2.320903	0.085139	-3.684026	2.333984	0.128680	-3.599491
C	1.838724	5.251066	-1.919034	1.740780	5.328338	-1.927194
H	2.567566	5.544049	-2.688411	1.668917	5.635711	-2.983790
H	0.871477	5.710162	-2.183657	0.938545	5.838590	-1.375962
H	2.159786	5.703488	-0.965814	2.703069	5.716818	-1.549185
C	-1.829883	5.254710	1.918815	-1.956280	5.201818	1.940903
H	-0.861495	5.711361	2.183487	-1.022530	5.699562	2.253044
H	-2.149998	5.708310	0.965834	-2.244230	5.637480	0.969866
H	-2.557907	5.549121	2.688424	-2.734624	5.468920	2.670163
C	-2.455160	0.178981	2.590182	-2.348639	0.111540	2.670451
H	-2.323394	0.088803	3.681491	-2.195872	0.034148	3.760089
H	-3.529740	0.030632	2.386208	-3.420728	-0.075598	2.486299
H	-1.896501	-0.634831	2.106069	-1.770542	-0.685879	2.182147
H				-2.274723	-3.407384	-5.587982
Cl				-1.365777	-3.651742	-6.487480

Table S5. Coordinates of $Br_2(CH_3)_4$BODIPY optimized structures in excited states for florescence

atom	neutral			acidic		
	X	Y	Z	X	Y	Z
B	0.001105	-1.418901	0.000061	-0.006632	-1.387249	0.156720
N	1.266992	-0.488029	0.000349	1.259345	-0.472239	0.121664
C	2.549269	-0.945837	0.000162	2.541606	-0.926234	0.192336
C	3.367283	0.186911	0.000115	3.360625	0.205028	0.107499
C	2.518175	1.400531	-0.000114	2.516484	1.410913	-0.019517
C	1.232634	0.919812	0.000211	1.229375	0.932571	-0.004058
C	-0.032083	1.627186	0.000178	-0.031889	1.639053	-0.075348
C	-1.213639	0.916925	0.000005	-1.215659	0.937162	0.000333
C	-2.570254	1.393775	-0.000392	-2.569969	1.416504	-0.015599
C	-3.356069	0.241065	-0.000052	-3.358646	0.271888	0.109061
C	-2.521895	-0.911735	0.000302	-2.529033	-0.880558	0.196224
N	-1.233578	-0.490422	0.000405	-1.237908	-0.466428	0.129435
F	0.025049	-2.210799	-1.137379	0.009394	-2.219284	-0.993885
F	0.025098	-2.211448	1.137011	0.013952	-2.185893	1.291325
C	2.908684	-2.393342	-0.000078	2.909605	-2.366028	0.317432
H	2.487716	-2.899898	0.882263	2.410356	-2.823161	1.185140
H	2.490288	-2.899088	-0.884141	2.590384	-2.936589	-0.569868
H	3.999424	-2.520053	0.001407	3.996166	-2.477110	0.429013
B	5.216338	0.213771	-0.000053	5.208037	0.229785	0.137103
C	3.001106	2.807915	-0.000149	2.999915	2.814144	-0.130111
H	3.630381	3.015684	-0.882808	3.653371	2.944451	-1.009799
H	2.165899	3.520669	-0.003166	2.165327	3.521945	-0.219669
H	3.625333	3.017423	0.885731	3.598221	3.102533	0.751347
C	-2.985131	2.826351	0.000143	-2.982083	2.844974	-0.133186
H	-2.587225	3.363857	-0.878617	-2.611144	3.297692	-1.069513
H	-4.079753	2.920907	-0.015051	-4.076400	2.942637	-0.123016
H	-2.613115	3.356080	0.895006	-2.581160	3.452098	0.697599
B	-5.239158	0.186789	-0.000077	-5.239994	0.227292	0.155149
C	-2.900822	-2.349386	0.000019	-2.918854	-2.309722	0.320926
H	-3.991745	-2.466407	0.003233	-4.006340	-2.409403	0.425536
H	-2.483224	-2.859462	-0.883815	-2.594465	-2.881964	-0.565314
H	-2.477509	-2.861247	0.880011	-2.422766	-2.773577	1.188627
H	-0.042049	2.714808	0.000133	-0.038670	2.722233	-0.174689
H				0.121005	-4.009960	-1.299889
Cl				0.246555	-5.274053	-1.586601

Table S6. Coordinates of $Zn[Br_2(CH_3)_4dpm]_2$ optimized structures in excited states for florescence

atom	neutral			acidic		
	X	Y	Z	X	Y	Z
Zn	0.101091	-0.002187	-0.050135	-0.128239	-0.005936	0.085054
N	-0.220975	0.027006	1.971939	-1.158937	-1.382696	-0.990218
C	-1.428956	0.055575	2.616020	-2.118502	-1.198746	-1.906463
C	-1.162601	0.065982	3.975330	-2.504757	-2.466449	-2.428004
C	0.322424	0.041846	4.165456	-1.757196	-3.461840	-1.807621
C	0.834914	0.018473	2.889473	-0.905758	-2.752287	-0.895587
C	2.253828	-0.010710	2.522153	0.037009	-3.324209	-0.030952
C	2.805028	-0.034659	1.252707	0.901889	-2.724603	0.885380
C	4.220322	-0.062803	0.948209	1.828339	-3.417018	1.741759
C	4.285956	-0.079701	-0.442531	2.449141	-2.413429	2.471062
C	2.967690	-0.062762	-0.974667	1.911975	-1.150877	2.064983
N	2.082047	-0.035804	0.048150	0.990864	-1.349263	1.117458
N	-0.868979	-1.498959	-1.007179	0.964764	1.283098	-1.066980
C	-0.741569	-2.825257	-0.867628	1.883324	0.958848	-2.028910
C	-1.666499	-3.476519	-1.734764	2.367751	2.150580	-2.543988
C	-2.385154	-2.511581	-2.430842	1.687519	3.281017	-1.838292
C	-1.864678	-1.258841	-1.956729	0.838400	2.670962	-0.944026
C	-2.267790	0.014709	-2.373076	-0.063598	3.343140	-0.005080
C	-1.817083	1.278678	-1.982689	-0.936723	2.765789	0.900969
C	-2.299384	2.534456	-2.489660	-1.811032	3.483530	1.804375
C	-1.551438	3.493964	-1.820183	-2.489612	2.491655	2.507944
C	-0.644401	2.833936	-0.935008	-2.052994	1.215667	2.059013
N	-0.813311	1.510985	-1.039585	-1.120023	1.386104	1.093391
Br	-2.734293	0.070965	1.888431	-3.822964	-2.735577	-3.760926
Br	-2.818498	0.951973	1.231306	3.786176	-2.626167	3.789050
H	-2.850762	-0.820674	1.250863	0.106648	-4.413527	-0.079278
Br	-3.574083	0.094215	2.596397	3.634950	2.375243	-3.877187
Br	-2.384246	0.102707	5.368848	-3.789849	2.771650	3.853636
H	1.013274	0.044953	5.480381	-0.040103	4.431602	-0.030793
C	0.712255	-0.824710	6.091070	-2.640743	0.144473	-2.296058
H	2.104483	0.023079	5.368814	-2.413012	0.893694	-1.525300
H	0.745515	0.940255	6.069183	-2.190869	0.481506	-3.246404
H	5.329698	-0.070965	1.945817	-3.730213	0.115063	-2.449139
C	5.269800	-0.946686	2.616707	-1.837085	-4.935316	-2.047405
H	6.309056	-0.098219	1.447568	-1.614325	-5.178757	-3.099806
H	5.306373	0.825597	2.591058	-1.134760	-5.499067	-1.418071
H	5.865921	-0.118758	-1.482767	-2.851487	-5.316664	-1.842846
C	2.539368	-0.070447	-2.403262	2.046375	-4.894600	1.822076
H	3.406305	-0.101598	-3.076030	1.153261	-5.416027	2.206195
H	1.899813	-0.942699	-2.623784	2.277855	-5.327176	0.835002
H	1.946123	0.827994	-2.647427	2.882364	-5.129426	2.495486
C	0.219130	-3.465856	0.078426	2.296539	0.197609	2.575167
H	0.688000	-4.351074	-0.377426	2.352864	0.199509	3.674848
H	1.007703	-2.761681	0.377745	3.295787	0.487590	2.206550
H	-0.296994	-3.812553	0.990848	1.574198	0.961574	2.257266
C	-1.868005	-5.348240	-1.895808	2.219272	-0.452812	-2.386559
H	-3.451279	-2.743863	-3.452655	3.013172	-0.483090	-3.145291
H	-3.364378	2.739511	-3.520001	2.566916	-1.018685	-1.506964
H	-3.074183	2.312638	-4.494810	1.343794	-0.985783	-2.792687
C	-4.314360	2.263810	-3.225306	1.931043	4.721964	-2.104598
H	-3.559493	3.810306	-3.671333	1.712228	4.974819	-3.157242
H	-1.675950	5.370368	-2.012000	1.316137	5.366028	-1.463672
H	0.340884	3.471185	-0.012627	2.991231	4.984240	-1.940304
C	-0.169808	3.955699	0.837564	-1.925253	4.966872	1.917330
H	1.047484	2.729623	0.384137	-0.961461	5.431484	2.192677
H	0.909847	4.259505	-0.529867	-2.238324	5.426879	0.962878
H	-4.257620	-3.376275	-3.045762	-2.662666	5.249385	2.681885
C	-3.049243	-3.274511	-4.332362	-2.487022	-0.133769	2.522994
H	-3.904181	-1.806740	-3.805108	-1.651566	-0.685352	2.989239
H	2.953913	-0.013718	3.356288	-3.296835	-0.056846	3.260263
H	-3.059960	0.025140	-3.125606	-2.843273	-0.750008	1.679755
H				-2.201844	-1.836540	-5.551451
Cl				-1.303011	-1.295193	-6.313722

Table S7. Coordinates of $Cd[Br_2(CH_3)_4dpm]_2$ optimized structures in excited states for florescence

atom	neutral			acidic		
	X	Y	Z	X	Y	Z
Cd	0.084295	0.007254	-0.160131	-0.179310	-0.000137	0.134586
N	0.916532	-0.077270	1.947945	-1.221343	-1.597254	-1.016908
C	0.189622	-0.117840	3.108153	-2.188647	-1.465713	-1.931966
C	1.096252	-0.164660	4.152535	-2.505125	-2.752041	-2.456974
C	2.478688	-0.151053	3.575290	-1.704041	-3.704497	-1.838841
C	2.287103	-0.096729	2.212457	-0.891314	-2.950076	-0.922721
C	3.351217	-0.063813	1.201888	0.074321	-3.494259	-0.064048
C	3.274156	-0.003166	-0.179992	0.929289	-2.919955	0.879063
C	4.420748	0.029081	-1.067160	1.857752	-3.657552	1.699285
C	3.882244	0.090829	-2.349388	2.474867	-2.696236	2.484290
C	2.464608	0.095980	-2.260686	1.934478	-1.414082	2.147268
N	2.105801	0.039849	-0.957086	1.016592	-1.560240	1.189037
N	-1.433530	-1.544052	-0.644115	0.996189	1.483428	-1.098704
C	-1.289876	-2.874036	-0.688254	1.914251	1.192544	-2.071464
C	-2.547379	-3.470776	-0.995387	2.385077	2.400430	-2.558915
C	-3.494581	-2.466060	-1.145800	1.696016	3.504648	-1.823331
C	-2.768837	-1.243074	-0.921129	0.854305	2.863877	-0.939481
C	-3.315368	0.044257	-0.987322	-0.045431	3.525300	0.010761
C	-2.761207	1.317848	-0.822140	-0.940689	2.989375	0.923409
C	-3.492224	2.551529	-0.952323	-1.786324	3.771578	1.802903
C	-2.550275	3.545354	-0.726475	-2.514896	2.833638	2.530095
C	-1.287679	2.928595	-0.466726	-2.136083	1.528125	2.120156
N	-1.426334	1.600319	-0.524651	-1.191172	1.627718	1.156019
Br	-1.305070	-0.106946	3.124732	-3.809207	-3.089489	-3.788374
Br	-1.708562	0.807761	2.658579	3.808737	-2.976906	3.792896
H	-1.722116	-0.965590	2.572107	0.175990	-4.579258	-0.143400
Br	-1.684274	-0.154077	4.154786	3.646373	2.671084	-3.891071
Br	0.733319	-0.233113	5.969701	-3.807941	3.211347	3.859962
H	3.730189	-0.191405	4.373770	-0.001252	4.613287	-0.012953
C	3.779476	-1.106835	4.990158	-2.787984	-0.154222	-2.319217
H	4.623760	-0.158830	3.738437	-2.560610	0.620738	-1.573079
H	3.778766	0.655850	5.080657	-2.400358	0.185258	-3.295711
H	5.852823	0.002802	-0.648957	-3.881576	-0.232501	-2.418750
C	6.095168	-0.909355	-0.074680	-1.708426	-5.179823	-2.081953
H	6.519283	0.033853	-1.522505	-1.537510	-5.405410	-3.147611
H	6.106916	0.862207	-0.002796	-0.935588	-5.702312	-1.501781
H	4.860097	0.156918	-3.968718	-2.683067	-5.623591	-1.817200
C	1.462458	0.141732	-3.364571	2.083588	-5.136289	1.695569
H	1.951467	0.274905	-4.338484	1.174279	-5.687241	1.988866
H	0.868602	-0.789346	-3.409858	2.379505	-5.501149	0.698109
H	0.748236	0.972053	-3.223308	2.880183	-5.410166	2.401179
C	0.003098	-3.575550	-0.433063	2.313324	-0.097329	2.738519
H	0.184014	-4.355406	-1.189141	2.307339	-0.143760	3.839050
H	0.847652	-2.871707	-0.448448	3.336319	0.190421	2.440696
H	-0.006382	-4.081906	0.547777	1.622966	0.696058	2.418827
C	-2.851420	-5.328104	-1.164809	2.260096	-0.207782	-2.463531
H	-4.941014	-2.639579	-1.482118	3.055045	-0.215666	-3.221777
H	-4.945104	2.708345	-1.273274	2.613489	-0.793672	-1.598451
H	-5.185946	2.311980	-2.274045	1.389590	-0.737843	-2.885913
C	-5.583321	2.175875	-0.548920	1.926377	4.953211	-2.058169
H	-5.234888	3.768237	-1.257751	1.702136	5.226917	-3.104533
H	-2.838012	5.413860	-0.746860	1.309107	5.578940	-1.401862
H	0.003324	3.613870	-0.162997	2.985249	5.220220	-1.892362
C	-0.023178	4.087574	0.833509	-1.836256	5.261255	1.874397
H	0.844193	2.906018	-0.186952	-0.854010	5.691858	2.139473
H	0.202891	4.418123	-0.888655	-2.128934	5.708401	0.907431
H	-5.429904	-3.330108	-0.775292	-2.561741	5.595640	2.629447
C	-5.065204	-3.077994	-2.486952	-2.629239	0.213898	2.624908
H	-5.494012	-1.690336	-1.460988	-1.844383	-0.324745	3.187099
H	4.360013	-0.090485	1.611539	-3.488848	0.344365	3.295498
H	-4.383024	0.059626	-1.219470	-2.938358	-0.443581	1.793621
H				-2.282425	-2.043670	-5.584854
Cl				-1.442985	-1.411271	-6.344654
